# Energy‐Efficient Iodine Uptake by a Molecular Host⋅Guest Crystal

**DOI:** 10.1002/anie.202214039

**Published:** 2022-10-26

**Authors:** Xue Yang, Chunyang Li, Michel Giorgi, Didier Siri, Xavier Bugaut, Bastien Chatelet, Didier Gigmes, Mehdi Yemloul, Virginie Hornebecq, Anthony Kermagoret, Sophie Brasselet, Alexandre Martinez, David Bardelang

**Affiliations:** ^1^ Aix Marseille Univ CNRS ICR AMUTech Marseille France; ^2^ School of Materials Science and Engineering & Material Corrosion and Protection Key Laboratory of Sichuan Province Sichuan University of Science & Engineering Zigong 643000 P. R. China; ^3^ Aix Marseille Univ CNRS Centrale Marseille iSm2 AMUTech Marseille France; ^4^ Aix Marseille Univ CNRS, Centrale Marseille, FSCM Spectropole Marseille France; ^5^ Université de Strasbourg Université de Haute-Alsace CNRS LIMA UMR 7042 67000 Strasbourg France; ^6^ Aix Marseille Univ CNRS MADIREL AMUTech Marseille France; ^7^ Aix Marseille Univ CNRS Fresnel AMUTech Marseille France

**Keywords:** Cucurbituril, Iodine Adsorption, Porous Material, Supramolecular, Verkade Superbase

## Abstract

Recently, porous organic crystals (POC) based on macrocycles have shown exceptional sorption and separation properties. Yet, the impact of guest presence inside a macrocycle prior to adsorption has not been studied. Here we show that the inclusion of trimethoxybenzyl‐azaphosphatrane in the macrocycle cucurbit[8]uril (CB[8]) affords molecular porous host⋅guest crystals (**PHGC‐1**) with radically new properties. Unactivated hydrated **PHGC‐1** adsorbed iodine spontaneously and selectively at room temperature and atmospheric pressure. The absence of (i) heat for material synthesis, (ii) moisture sensitivity, and (iii) energy‐intensive steps for pore activation are attractive attributes for decreasing the energy costs. ^1^H NMR and DOSY were instrumental for monitoring the H_2_O/I_2_ exchange. **PHGC‐1** crystals are non‐centrosymmetric and I_2_‐doped crystals showed markedly different second harmonic generation (SHG), which suggests that iodine doping could be used to modulate the non‐linear optical properties of porous organic crystals.

## Graphical abstract

A hydrated and molecular porous host⋅guest crystal (**PHGC‐1**) made of cucurbit[8]uril and trimethoxybenzylazaphosphatrane can adsorb iodine spontaneously and selectively at room temperature and atmospheric pressure. The absence of a) heat for material synthesis, b) moisture sensitivity, and c) energy‐intensive steps for pore activation are very attractive attributes of this new generation of energy‐saving porous materials.

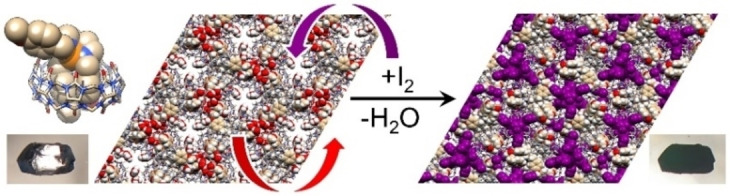

## Introduction

**Figure 1:**
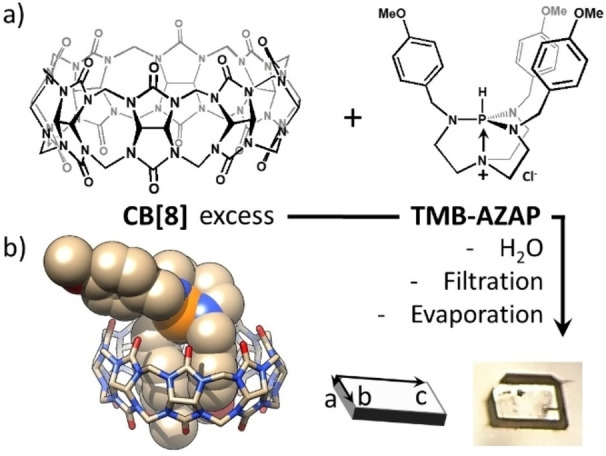
a) Structures of cucurbit[8]uril (CB[8]) and of trimethoxybenzyl‐azaphosphatrane (**TMB‐AZAP**). b) Single‐crystal structure of the corresponding 1 : 1 host⋅guest complex (hydrogen atoms removed for clarity; bottom right, photo of a **PHGC‐1** single crystal).

Porous materials are key compounds for academics and industry. Among the categories of porous materials, a rising class is that of Porous Organic Crystals (POCs).[^1^, ^2^, ^3^, ^4^, ^5^, ^6^, ^7^, ^8^, ^9^] While zeolites[^10^, ^11^] or hybrid materials such as Metal‐Organic Frameworks (MOFs)[^12^, ^13^, ^14^] contain inorganic compounds, POCs are purely organic materials. Besides Covalent Organic Frameworks (COFs), which are characterized by polymerized strong covalent bonds,[^15^, ^16^, ^17^] most POCs are based on the crystallization of small molecules for which supramolecular interactions and packing effects govern the structure of the final assembly.^[18]^ Among the POC materials, those with intrinsic porosity are mainly obtained using macrocyclic or container‐shape compounds,[^2^, ^4^, ^6^, ^19^] while those with extrinsic porosity rely on particular patterns creating hollow spaces.[^20^, ^21^, ^22^, ^23^, ^24^, ^25^, ^26^, ^27^, ^28^, ^29^, ^30^, ^31^, ^32^, ^33^] In this context, macrocycle porous crystals made of cucurbit[*n*]urils (CB[*n*])[^34^, ^35^, ^36^, ^37^, ^38^] such as CB[6] or CB[8] (Figure 1a) have a particular place.

Indeed, with their quite rigid shape and preorganized cavity, CB[*n*] are endowed with intrinsic porosity,^[39]^ and when properly crystallized, extrinsic porosity.[^40^, ^41^] That is well illustrated by CB[6] crystals capable of CO_2_ adsorption both inside macrocycles and in tubular channels formed by the outer surface of CB[6].^[41]^ However, despite important progresses obtained with POC based on macrocycles,[^39^, ^40^, ^41^, ^42^, ^43^, ^44^, ^45^, ^46^, ^47^, ^48^, ^49^, ^50^, ^51^, ^52^, ^53^, ^54^, ^55^] there is no report to our knowledge about the use of guest molecules inside macrocycles to modulate POC porosity by forming 1 : 1 host⋅guest complexes. Inclusion of guest molecules can beneficially reduce porous space (i.e. when adsorption is pursued instead of storage), thereby impacting pore size, shape and polarity. These modifications can in turn dramatically improve the *selectivity of adsorption*,[^28^, ^56^] a parameter as important as capacity when addressing gas capture in real conditions (i.e. adsorption from a mixture or from filled pores, water often being a problem for material stability or behaving as competitor).

## Results and Discussion

**Figure 2:**
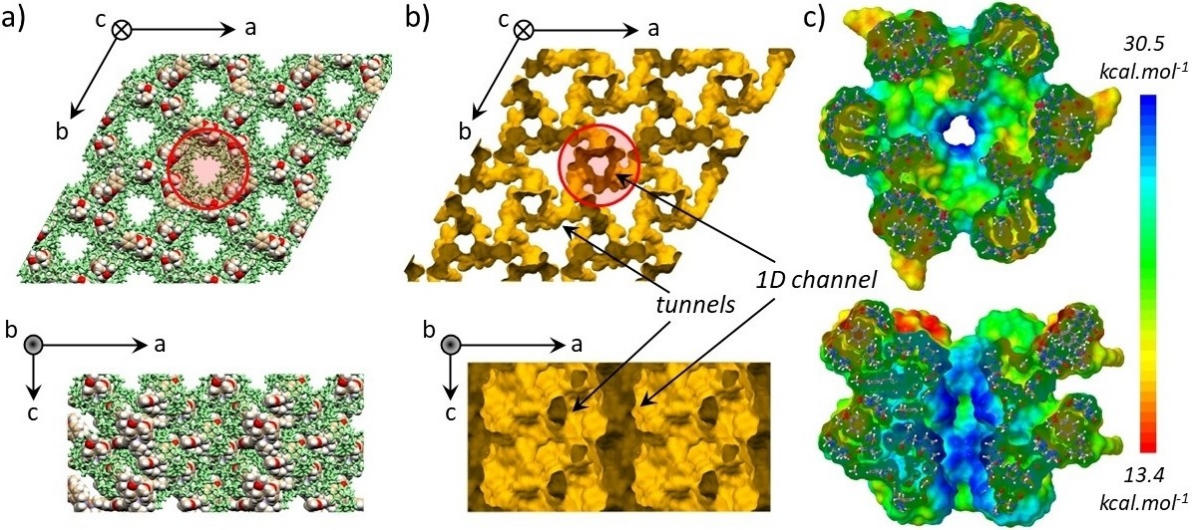
a) Crystalline packing of the **PHGC‐1** crystals showing 1D channels along axis c (hosts shown in green as cylinder and guests as space‐filling representations to highlight the presence of the guests in the host⋅guest network; water molecules removed for clarity). b) Shape of the channels viewed down crystallographic axes c (top) and b (bottom) illustrating the presence of 1D channels connected by twisted tunnels. c) Cut view of the electrostatic potential surface of the channels modeled considering 24 host⋅guest complexes (24+ overall charge). The channels are characterized by a positive electrostatic surface as well as the twisted tunnels, but to a lesser extent. Note that chloride counter‐anions must also partially shield this positive surface.

Here we describe our discovery of a new POC of *host*⋅*guest* type (molecular Porous Host⋅Guest Crystals, **PHGC‐1**, Figure 2) in which the guest, trimethoxybenzyl‐azaphosphatrane (**TMB‐AZAP**), changes CB[8] packing compared to a landmark porous CB[8] crystal,^[57]^ remodelling the channels while preserving both stability and porosity. We next found that these crystals, replete with channels filled by water molecules, could adsorb iodine molecules at the *solid–gas* or *solid–liquid interface*. There is a growing research effort to find materials able to capture iodine for detection, uptake and treatment of the dangerous isotopes ^129^I (*t*_1/2_=1.6×10^7^ years) and ^131^I (*t*_1/2_=8.02 days) produced (i) in nuclear power plants, and (ii) after nuclear accidents (i.e. 1979, 1986, 2011) where ^129^I and ^131^I are released presenting major concerns for health (mainly thyroid cancer).^[58]^ Storing iodine could also be of interest for satellite propulsion, a recently demonstrated technology,^[59]^ or for electron conducting materials.[^60^, ^61^] While I_2_ storage requires high capacity, I_2_ capture needs high selectivity in real conditions. Most porous materials adsorb water from ambient humidity posing several issues for I_2_ adsorption (material stability, uptake competition) but the problem of I_2_/H_2_O selectivity has only been seldom studied[^62^, ^63^, ^64^, ^65^, ^66^] compared to the capture of other gases where the field is more mature (i.e. CO_2_).^[67]^ Although not featured by high sorption capacity compared to other POC based on macrocycles for iodine capture,[^68^, ^69^, ^70^] **PHGC‐1** are endowed with several key advantages: (i) their growth from water requires almost no energy, (ii) they are hydrated so insensitive to moisture, (iii) they capture I_2_ spontaneously and *selectively* (compared to water) at room temperature and (iv) they do not require energy‐intensive pore activation, contrary to most porous materials,[^71^, ^72^, ^73^, ^74^, ^75^] POC,[^2^, ^3^, ^8^, ^15^, ^28^, ^43^, ^44^, ^45^, ^46^] or adaptive non‐porous crystals.[^48^, ^50^, ^52^, ^55^]

Azaphosphatranes are the protonated counterpart of pro‐azaphosphatranes also named Verkade's superbases.[^76^, ^77^] These phosphorus derivatives are robust hydrophobic cations and have been used as organocatalysts or as motifs to build self‐assembled cages for anion recognition.[^78^, ^79^, ^80^] Investigating the inclusion of **TMB‐AZAP** with various amounts of CB[8], we found by ^1^H NMR (Figure S1) evidence for formation of a 1 : 1 complex. Moreover, aromatic resonances were split into two sets of signals, one set upfield shifted[^81^, ^82^] and integrating twice that of the second, downfield shifted set of signals (Figure S1). COSY experiments confirmed the occurrence of two different sets of aromatic resonances (Figure S2) and led us to postulate the existence of a CB[8]⋅**TMB‐AZAP** 1 : 1 complex in which two aromatic groups are immersed in the host cavity, leaving the 3^rd^ *p*‐methoxy‐benzyl group bulk exposed. Competition with dimethyl‐viologen enabled to determine a binding constant of **TMB‐AZAP** toward CB[8], *K*_a_=1.45×10^7^ M^−1^ (Figure S3). The excess host needed to saturate the guest remains unclear even if we recently reported a similar effect with CB[10].^[83]^

**Figure 3:**
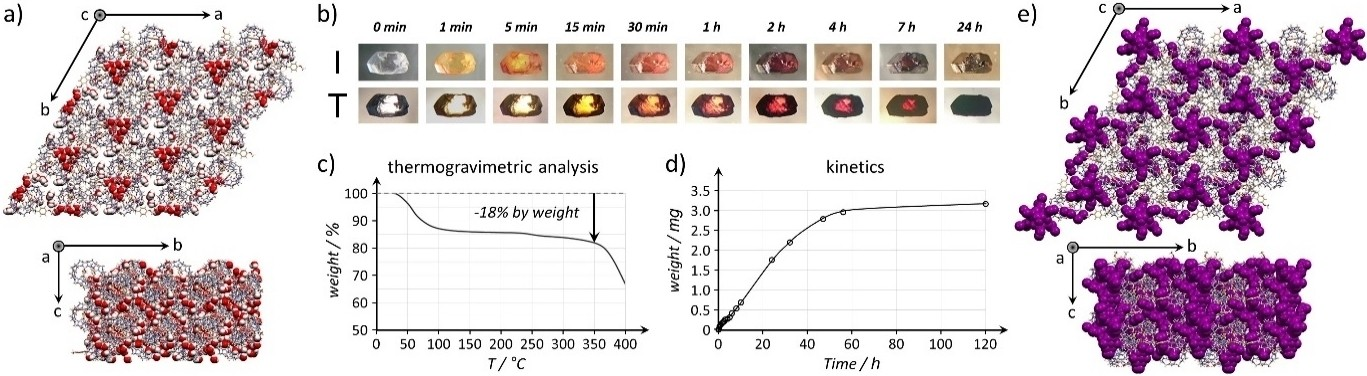
a) Crystal structure of hydrated **PHGC‐1**. b) Monitoring of guest exchange at the gas‐solid interface of **PHGC‐1**, iodine molecules progressively replacing water molecules in the crystal lattice (I: incident light; T: transmitted light). c) Representative thermogram of **PHGC‐1** crystals, and d) kinetics of iodine adsorption by 8.3 mg of **PHGC‐1** crystals. e) Crystal structure of **PHGC‐1‐I_2_** with channels filled by iodine molecules that are mainly disordered.

Slow evaporation of aqueous solutions of the complex repeatedly afforded rather large (≈1–7 mm) colorless single crystals suitable for single crystal X‐ray diffraction (Figure 1b, Figure 2, CCDC‐2143333).^[84]^ The corresponding structure showed the guest engulfed in the CB[8] cavity by two of its *p*‐methoxy‐benzyl arms (Figure 1b), the 3^rd^ guest appended group close to one of the CB[8] carbonyl rims. There are several C−H⋅⋅⋅O hydrogen bonds[^85^, ^86^] involving the side‐arms and the phosphatrane skeleton toward oxygen atoms of the host and the two included phenyl groups are slightly shifted and too far away for ideal dispersive stacking interactions. Interestingly, the hydrogen atom carried out by the phosphorus atom is not engaged in strong H bonding with the host crown contrary to nearby hydrogen atoms of the phosphatrane moiety. Crystal packing (Figure 2a) reminds that of CB[8] crystallized from aqueous formic acid,^[57]^ but the non‐included *p*‐methoxy‐benzyl group significantly changed it by additional interactions with neighboring CB[8] (Figure S4) thereby changing both the structure and the nature of the channels. Indeed, while the known channel structure showed rather uniform 1D channels (Figure S5),^[57]^ these crystals are featured by slightly corrugated 1D channels connected by small twisted tunnels, filled by water molecules (Figure 2b). The incorporation of azaphosphatrane also changed the electrostatic nature of the channels by means of the positive charge of the guest. Molecular modelling (Supporting Information) enabled to map the electrostatic potential surface of the channels (Figure 2c). The surface of the channels is electropositive, as that of the connecting tunnels which are however less so. Beside some structural water molecules surrounding the 1 : 1 host⋅guest complexes, **PHGC‐1** crystals contain a *large amount of unlocated water molecules* (and Cl^−^ anions) in the channels which we preliminary assigned to high disordering, but another explanation may account for this result (see below). For one CB[8]⋅**TMB‐AZAP** complex, there are 6.5 water molecules plus unassignable electron densities accounting for approximately 15–20 additional water molecules (≈24±2 water molecules per host⋅guest complex, or ≈18.5 % by weight). The total number of water molecules per asymmetric unit (1 **TMB‐AZAP** and 1 CB[8]) is in good agreement with results obtained by elemental analysis (22 water molecules per host⋅guest complex, or 17.2 % by weight). Further analyses by TGA confirmed this amount of water with a mass loss of ≈18 % between 30 °C and 350 °C corresponding to approximately 23 water molecules (Figure 3c and Figure S6). Activation by placing the single crystals under high vacuum for 8 hours showed numerous cracks preventing full XRD characterization. Nevertheless, unit cell parameters could be measured and were identical to those of the initial phase. We observed that this material could rapidly adsorb iodine molecules but we next focused on *unactivated* crystals since these had the property to adsorb iodine vapors at room temperature even if the channels are *totally filled by water molecules* (Figure 3).

Placing the *unactivated* crystals (Figure 3a) in a saturated atmosphere of iodine at room temperature resulted in a rapid crystal coloration from colorless to yellow, red, and finally black (Figure 3b) suggesting capture of I_2_ in the **PHGC‐1** crystals with retention of the crystal structure. Placing 12.02 mg of colorless **PHGC‐1** crystals in a saturated atmosphere of I_2_ for 4 days resulted in 17.40 mg of black crystals (30.9 % weight gain). Elemental analysis confirmed a >30 % weight of iodide adsorbed (36.3 %). We next placed unactivated **PHGC‐1** crystals in an iodine‐saturated hexane solution for one week before analyzing the crystals again by SCXRD (CCDC‐2143334^[84]^).^[87]^ The structure appeared to be very close to that of initial **PHGC‐1** crystals considering the host⋅guest framework, but was otherwise replete with iodine molecules in place of water (Figure 3e). Unit cell parameters remained quite similar for a and b while we noted a slight contraction of ≈1.26 % along the channels’ axis (*c*=17.0157(1) Å for hydrated crystals, *c*=16.8020(2) Å for halogenated crystals). Besides the large number of iodine molecules, only one water molecule remained and one ordered I_2_ molecule was found located within the host⋅guest framework. The asymmetric unit accounted for 4 to 6 iodine atoms per host⋅guest complex which represented an approximate 21–29 % iodine capture by weight. These experiments unambiguously showed the accessible porosity of hydrated **PHGC‐1** crystals and its relevance for gas adsorption. All techniques converged toward a ≈30 % weight of iodide absorbed which is comparable to the capacity of some zeolites.^[88]^ Iodine uptake capacities of POC based on macrocycles alone clearly exceed the performances of **PHGC‐1**,[^68^, ^69^, ^70^] but adsorption capacities are recorded at 75 °C and these materials are activated prior to measurements. Beside high recording temperature, it is important to note that material activation is an energy‐intensive step, usually followed by unwanted water uptake (for most porous materials)[^71^, ^89^] thereby limiting their capacity in real conditions, and so restricting their use.^[90]^ Conversely, **PHGC‐1** crystals are stable, hydrated, and still remarkably adsorb I_2_ *spontaneously* at *room temperature* and *ambient pressure without activation*, and *selectively* (compared to water) properties that are difficult to reconcile in a unique material.

Besides CH⋅⋅⋅O hydrogen bonds, there are many C−H⋅⋅⋅I hydrogen bonds and several C=O⋅⋅⋅I halogen bonds[^29^, ^91^, ^92^] (Figure S7). The C−H⋅⋅⋅I interactions dominate (distances H⋅⋅⋅I in the range 2.62–3.06 Å),^[93]^ but some iodine atoms are also engaged in quite strong C=O⋅⋅⋅I halogen bonds with CB[8]^[94]^ (O⋅⋅⋅I distances between 2.69 Å and 3.11 Å, ≈11 to 23 % smaller than the sum of corresponding van der Waals radii (3.5 Å)). These interactions may be part of the driving force of guest exchange in **PHGC‐1**. Iodine desorption occurs but only very slowly at room temperature (for comparison, samples heated at 300 °C only loose around 20 % by weight), a useful property if I_2_ must remain stored (i.e. capture of a dangerous radioiodine). However, placing **PHGC‐1‐I_2_** crystals in pentane or water showed slow iodine release over time, so the host and guest can in principle be recycled to produce again **PHGC‐1**.

**Figure 4:**
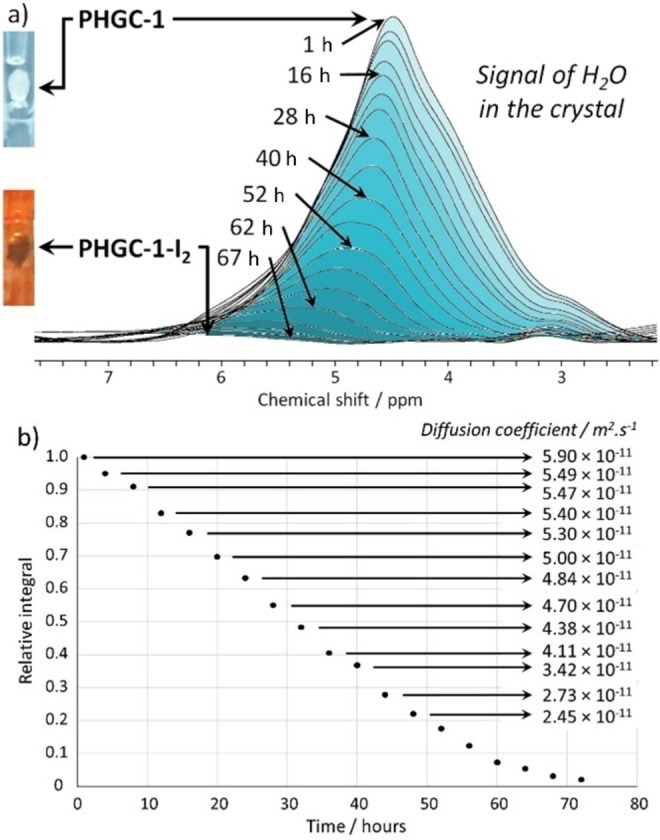
a) ^1^H NMR spectra of a **PHGC‐1** crystal slowly turning to **PHGC‐1‐I_2_** as a function of the diffusion of iodine molecules in the crystalline matrix over time (insets: photos of the bottom of the NMR tube showing the immobilized crystal, before and after iodine diffusion). b) Evolution of the signal integral and of the diffusion coefficient of water over time.

^1^H NMR spectra of a **PHGC‐1** single crystal recorded at 600 MHz on a liquid state spectrometer showed a very large peak featured by several shoulders assigned to water molecules experiencing different kinds of environment in the crystal lattice (Figure 4a). These are assigned to water molecules inside the channels or in the small tunnels connecting the channels (Figure 2b). DOSY NMR allowed to determine a diffusion coefficient, identical for all shoulders *D*=1.7×10^−11^ m^2^ s^−1^, about two orders of magnitude lower than that of liquid water (*D*=2.3×10^−9^ m^2^ s^−1^). Increasing the delay time showed that the water molecules are freely (though slowly) diffusing in the crystalline matrix and are not restricted to small, isolated pockets. This mobility of water molecules can explain the impossibility for SCXRD to locate them precisely. Placing one **PHGC‐1** crystal in the detection zone of an NMR tube (Figure 4 inset) with an iodine crystal placed at the bottom of the tube allowed to monitor the iodine capture by **PHGC‐1** and simultaneous water expulsion over time (Figure 4a). The initial large signal gradually decreased over time up to about 67 hours. Following the evolution of the diffusion coefficient (Figure 4b) with time showed a rather surprising and abrupt increase (Figure S8) from *D*=1.70×10^−11^ m^2^ s^−1^ to *D*=5.90×10^−11^ m^2^ s^−1^ after 1 hour exposure to iodine vapors, before slowly decreasing to *D*=2.45×10^−11^ m^2^ s^−1^ for the last amenable spectrum at 48 hours. The absence of two populations ascribed to “tightly” and “loosely” bound water, replaced by I_2_ molecules at expected different speed suggests that water molecules bound to the matrix exchange quite rapidly with unbound water. The initial abrupt increase of the *D* value remains difficult to explain but could be due to (i) local temperature increases if the adsorption of iodine is exothermic, (ii) iodine replacing immobilized water, the later reaching the bulk of the channels and becoming more mobile, or (iii) I_2_ reaching first the more encumbered spaces so chasing first the more immobilized water. The following decrease in water mobility could be due to gradually restricted available space caused by iodine molecules filling the channels over time.

**Figure 5:**
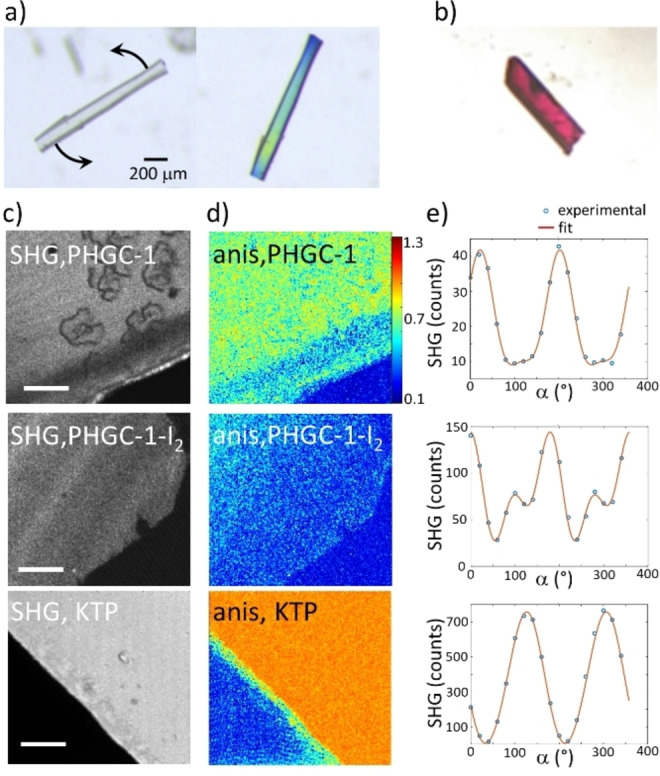
Single crystal of **PHGC‐1** viewed a) under polarized light and b) example of a **PHGC‐1‐I_2_** crystal studied by SHG microscopy. SHG image areas c) are shown for both crystals, together with a reference KTP crystal (note that measurements are taken at different incident intensities, scale bar: 20 μm). Anisotropy (“anis”) values d) obtained from the SHG polarization responses (with α the rotating polarization angle) depicted in e) at a centre region of the crystals.

Finally, observations of **PHGC‐1** crystals under a polarized microscope showed coloured crystals when their orientation was changed with respect to the angle of the incident polarized light (Figure 5a). We surmised a form of dichroism possibly due to the inherent anisotropy in crystal packing (*R*3, non‐centrosymmetric space group). Most compounds pack in crystal structures such as compensating their dipolar moments, which is not the case of **PHGC‐1**.^[95]^ The resulting macroscopic polar property is a requisite for a number of technological applications such as non‐linear optics (second harmonic generation abbreviated SHG), piezoelectricity or ferroelectricity.[^96^, ^97^] The generation of second harmonics was thus investigated on hydrated (**PHGC‐1**) and iodated (**PHGC‐1‐I_2_**) crystals (Figure 5c and Figure S9).

While the SHG response was weak but significant for the hydrated crystals (nonlinear coefficient estimated to be about 0.04 times the coefficient of KTP (Potassium Titanyl Phosphate)), the presence of iodine showed markedly increased intensities with altered signals (about 13 times higher SHG intensity). This effect is assignable to (i) the crystals becoming highly coloured (possible resonance at the excitation wavelength of the laser at 800 nm, see UV/Vis spectrum of **PHGC‐1‐I_2_** Figure S10), (ii) the presence of polarizable iodine molecules in the non‐centrosymmetric host⋅guest matrix, or (iii) defects in the crystal structure. Moreover, polarized SHG evidence the presence of higher orders in the polarization responses, which is correlated with a reduction of the anisotropy strength in the host⋅guest matrix (the SHG anisotropy coefficient is reduced by a factor of 1.4 in **PHGC‐1‐I_2_** as compared to **PHGC‐1**, Figure 5d, 5e). This shows a modification of the polarizability tensorial structure/anisotropy, which could be induced by the presence of iodine molecules. We are not aware of results showing modulated second harmonic generation by iodine doping in porous crystals.

## Conclusion

In conclusion, we have shown that the simple addition of a suitable guest to CB[8] in water before crystallization enabled to get a new porous (molecular) host⋅guest crystal type, **PHGC‐1**, with markedly new properties.

Not only the azaphosphatranes change the shape of the channels of known CB[8] crystals, but they also bring a permanent positive charge to the complexes, thereby rendering the surface of the channels electrostatically positive. This very simple method enabled to get a new type of porous organic crystals (POC) with preserved crystallinity, stability and porosity, even if the channels are totally filled by water molecules. This was well‐illustrated by the exchange of water molecules by iodine molecules at the solid–gas interface, **PHGC‐1** behaving as a crystalline sponge for I_2_. While most previous porous materials (PM) have guests as solvent that must be removed (activation) prior to iodine capture (see below), **PHGC‐1** can be used as‐prepared, the iodine uptake working by direct guest exchange with water of crystallization.

$$\text{-}\mathrm{Most}\;\mathrm{previous}\;\mathrm{materials}=\;\mathrm{guest}\cdot \mathrm{PM}\;\left(\Delta ,\;\mathrm{vacuum}\right)\to \mathrm{PM}\to I{}_{2}\cdot \mathrm{PM},$$

$$\text{-}\mathrm{This}\;\mathrm{material}=H{}_{2}O\cdot \mathrm{PM}\;\left(\mathrm{no}\;\Delta ,\;\mathrm{vacuum}\right)\to I{}_{2}\cdot \mathrm{PM}.$$

The numerous C−H⋅⋅⋅I and C−O⋅⋅⋅I interactions are probably in part responsible for this surprising guest exchange in a water‐saturated crystal. While iodine capture usually requires energy‐intensive material activation,[^68^, ^69^, ^70^, ^88^, ^98^] **PHGC‐1** could be advantageous due to (i) the absence of energy devoted to its synthesis, (ii) its moisture insensitivity, (iii) its absence of energy‐intensive activation step, (iv) the adsorption possible at room temperature and (v) the I_2_/H_2_O selectivity. The dynamics of water motion inside the crystals was probed by DOSY NMR as a function of water replacement by iodine molecules. Finally, the **PHGC‐1** crystals were shown to be dichroic, a property most likely caused by their non‐centrosymmetric nature and probed by SHG which revealed non‐linear optical properties largely impacted by the presence of iodine molecules in the crystalline matrix. The possibility to modulate the nature and the shape of the channels in CB‐based POC by means of guest inclusion before crystallization, and possibilities for guest adsorption add a new dimension to the design of tailor‐made porous organic crystals. To the best of our knowledge, there is no material with this combination of features for porous materials (low‐energy synthesis, moisture insensitivity, no activation, I_2_ selectivity (compared to H_2_O), gas adsorption at room temperature and ambient pressure, and SHG). This should foster research efforts toward more sustainable porous materials with reduced carbon footprint.^[99]^ We are now exploring how adaptation of the guest structure impacts the channels shape (porous space), adsorption, and SHG properties.

## Conflict of interest

The authors declare no conflict of interest.

1

## Supporting information

As a service to our authors and readers, this journal provides supporting information supplied by the authors. Such materials are peer reviewed and may be re‐organized for online delivery, but are not copy‐edited or typeset. Technical support issues arising from supporting information (other than missing files) should be addressed to the authors.

Supporting Information

Supporting Information

Supporting Information

## Data Availability

The data that support the findings of this study are available from the corresponding author upon reasonable request.
