# Supplementary material for: Energy‐Efficient Iodine Uptake by a Molecular Host⋅Guest Crystal
Source: Angew Chem Int Ed Engl. 2022 Oct 26;61(49):e202214039. doi: 10.1002/anie.202214039 (PMC10092189; doi:10.1002/anie.202214039)
Supplement: Supplementary file 1 — Supporting Information [file ANIE-61-0-s002.pdf]

Angewandte Chemie
Eine Zeitschrift der Gesellschaft Deutscher Chemiker

Supporting Information

Energy-Efficient Iodine Uptake by a Molecular Host·Guest Crystal

X. Yang, C. Li, M. Giorgi, D. Siri, X. Bugaut, B. Chatelet, D. Gigmes, M. Yemloul, V. Hornebecq, A. Kermagoret, S. Brasselet, A. Martinez, D. Bardelang**

Supporting Information

Table of Contents

- **Experimental procedures**

1/ Chemical compounds -- S2

2/ NMR Measurements -- S2

3/ Molecular modelling -- S2

4/ Single-crystal X-ray diffraction -- S2

5/ Thermogravimetric analysis -- S2

6/ Elemental analysis -- S2

7/ Solid state UV-vis spectroscopy -- S2

8/ Polarized second harmonic generation (SHG) microscopy --- S3

- **Additional data**

9/ Preparation, NMR spectra and binding constant for the CB[8]•**TMB-AZAP** complex -------------- S4

10/ Figures and table from the CB[8]•**TMB-AZAP (PHGC-1)** crystal structure ------------------------- S8

11/ TGA analysis of **PHGC-1** -- S10

12/ Elemental analysis of **PHGC-1** and **PHGC-1•I_2** --- S10

13/ Figures and table from the CB[8]•**TMB-AZAP•I_2 (PHGC-1•I_2)** crystal structure ------------------- S11

14/ Variation of the diffusion coefficient of water as a function of I_2 diffusion --------------------------- S12

15/ Results of Second Harmonic Generation -- S13

16/ UV-vis spectrum of **PHGC-1•I_2** crystals -- S13

17/ Iodine uptake from solution by **PHGC-1** crystals -- S14

18/ References -- S14

- Experimental procedures

1/ Chemical compounds. Distilled water was used to grow single crystals and iodine, D_2O and Nylon membrane filters (450 nm) were purchased from commercial sources. CB[8] was prepared according to a previous paper.[1] **TMB-AZAP** was prepared according to the literature.[2]

***2/ NMR Measurements*.** NMR spectra were recorded on BRUKER Avance III nanobay – 300 or 400 spectrometers (^{1}H-NMR frequencies 300.13, and 400.13 MHz) at 300 K using D_2O as the solvent and a watergate sequence (water suppress) when necessary (potentially affecting signals and integrals near the signal suppressed). Acetone was also used when necessary, as a reference. Splitting patterns are indicated as follows: s, singlet; d, doublet; t, triplet; m, multiplet. The spectra of the water inside the crystal were recorded at 300 K on a BRUKER Avance III – 600 MHZ spectrometer, using the standard one pulse sequence with 16 scans and a sum of the acquisition time and the relaxation delay equal to 3 seconds. NMR self-diffusion experiments were recorded at 300 K, by a conventional pulse sequence, based on the stimulated echo and incorporating bipolar gradient pulses and an Eddy current delay (BPP-LED).[3] The shape of all gradient pulses was Smoothed Square and the LED delay was of 5 ms. The diffusion delay was set at 100 ms, the gradient strength, *g,* was linearly incremented in 32 steps from 2% to 98% of its maximum value with a constant duration of 4 ms, and 32 scans. The sum of the acquisition time and the relaxation delay was equal to 3 seconds. The diffusion coefficients were obtained using the dynamic center available on TopSpin Software.

***3/ Molecular modelling*.** The molecular electrostatic potential (MEP) was depicted on van der Waals surface with the Jmol software.[4] A structure containing 24 CB[8]•**TMB-AZAP**$^{1+}$ complexes, portion of a channel, was constructed based on the single-crystal X-ray structure without water. The atomic charges were calculated with Ampac 11[5] at the PM6[6] level of theory.

4/ Single-crystal X-ray Diffraction. Suitable crystals for CB[8]•**TMB-AZAP** and CB[8]•**TMB-AZAP•**I_2 were measured on a Rigaku Oxford Diffraction SuperNova diffractometer at 295 K at the CuKα radiation (λ=1.54184 Å). Data collection reduction and multiscan ABSPACK correction were performed with CrysAlisPro (Rigaku Oxford Diffraction). Using Olex2,[7] the structures were solved with ShelXT[8] using intrinsic phasing and ShelXL[9] was used for full matrix least squares refinements. For CB[8]•**TMB-AZAP** 6.5 mostly disordered water molecules were determined experimentally and were refined with partial occupation factors when necessary. A mask of solvent accounting more or less for 4300 Å^3 per unit cell was applied in the final stages of the refinement. For CB[8]•**TMB-AZAP•**I_2 5 iodine atoms were found in the asymmetric unit, extremely disordered over several sites and refined with partial occupation factors, as well as one full structural water molecule. Most of the H-atoms were found experimentally for CB[8]•**TMB-AZAP** except some of the azaphosphatrane and those of the water molecules that were introduced at geometrical positions. All H-atoms were introduced at geometrical positions for CB[8]•**TMB-AZAP•**I_2. In both cases their coordinates and Uiso parameters were constraint to 1.2Ueq (parent atoms) for the CH and CH_2 groups and to 1.5Ueq (parent atoms) for all the other groups.

5/ Thermogravimetric analysis. Thermogravimetric analysis was executed in a TGA 8000TM (PerkinElmer) apparatus. The tests were conducted at a rate of 5°C/min from 30°C to 800°C under inert atmosphere (Nitrogen flow rate: 20 mL/min) followed by a rate of 20°C/min from 800°C to 1000°C under air and holding for 7 minutes at 1000°C.

***6/ Elemental analysis*.** CHN elemental analyses were performed using an EA analyser, Flash EA 1112 series Thermo Finnigan driven by the Eager Xperience software (oven temperature: 970°C, gas: helium, flow rate: 140 mL/min, detector: catharometer).

***7/ Solid-state UV-vis spectroscopy*.** Diffuse reflectance measurements were performed in the 400-800 nm range, using a Varian 300 spectrophotometer equipped with an integrating sphere DRA-CA-30I. The crystals were placed in a Teflon sample holder. Several measurements were done to account for problems arising from a possible surface inhomogeneity and a mean value of the reflectance was used that was then converted in absorbance (A).

8/ Polarized second harmonic generation (SHG) microscopy. Polarized SHG setup. The light source was a tunable mode-locked Ti:Sapphire laser (Chameleon Ultra II, Coherent Inc.) operating at a wavelength of 800 nm, delivering 150 fs pulses (repetition rate 80 MHz) to an optical parametric oscillator (Compact OPO-Vis, Coherent Inc.). The excitation beam was sent to an inverted microscope (Eclipse Ti, Nikon Instruments Inc.) using a pair of galvonometric scan mirrors (6215H, Cambridge Technology Inc.) and a dichroic mirror (735 nm cutoff, Semrock). The excitation light was focused on the sample by a high numerical aperture objective (NA 1.15, 40x, Nikon Instruments Inc.) and the emitted light was collected in the back reflected direction by the same objective. The SHG signal at the wavelength 400 nm was recorded with photomultiplier tubes (PMTs, Hamamatsu R9110), and filtered using 400/10nm band-pass filters. Scanning and data acquisition was performed using an in-house LabVIEW (National Instruments Corp.) program. The image acquisition was performed with a pixel dwell time of 20 μs, over 100×100 pixels covering a field of view of about 50 to 100 μm size. Typical average powers at the focal plane were about 10 to 50 mW. To perform the polarized SHG measurements, a polarizing beamsplitter (PBS252, Thorlabs Inc.) was placed after the scanning mirrors to ensure that the input polarization was linearly polarized when reflected on the incident reflection dichroic mirror along its p or s polarization direction. An achromatic half-wave plate HWP (AQWP10M-980, Thorlabs Inc.) was placed after this dichroic mirror, mounted on a motorized rotational mount (PR50CC, Newport Corp.) to rotate the incident linear polarization by an increasing angle α with respect to the X axis (horizontal axis in the sample image). We recorded the image for each polarization state over the range of 0° to 180° with an α angle step of 10°, chosen to provide a signal to noise of the total signal above 5 to ensure sufficient precision (~ a few degrees) on the retrieved parameters (anisotropy and orientation, see below). The emitted nonlinear signal was detected in the reflected direction without any analyzer in the detection path.

Polarized SHG data analysis. The quantification of the polarized SHG responses follows the principle derived in Reference 10.[10] Briefly, each pixel of an image is a recorded stack of images, from which we retrieve the modulation of the signal as a function of the input polarizations angle α. At each pixel of the SHG image, the modulation can be expressed $I^{SHG}(\alpha) \propto 1 + A_2 \cos 2(\alpha - \varphi_2) + A_4 \cos 4(\alpha - \varphi_4)$. The anisotropy parameter A_2 is retrieved by circular projection of the intensity modulation onto the function $(\cos 2\alpha)$. The fourth order parameter A_4 is the signature of high order symmetry in the crystal. Both parameters depend strongly on both the symmetry of the crystal and its orientation in the measured sample. In this work we focused on A_2 which is a signature of the one-dimensional symmetry of the crystal projected in the sample plane. For each crystal region measured (typically 50x50 μm^2) a map of A_2 and intensity values is reported. Mean and standard deviation values were recorded over 6 crystal regions (taken on 4 different crystals) as plotted in Figure S8. The deviation visible from crystal to crystal is likely due to the different crystals off-plane orientations in the sample, nevertheless clear differences can be seen in the two crystals. In **PHGC-1**, $A_2 = 0.52 \pm 0.07$ and intensity values (in V) are of the order of $0.32 \pm 0.07\,V$, while in **PHGC-1-I$_2$** , $A_2 = 0.37 \pm 0.05$ and intensity values are of the order of $3.62 \pm 3.2\,V$. In comparison, values reported in a KTP crystal are $A_2 = 1.04 \pm 0.13$ and intensity (for an identical incident intensity) around $764\,V$. At last, the angle φ_2 corresponding to the phase of the measured intensity modulation was also retrieved, which corresponds to the in-plane orientation of the anisotropy response of the crystal. In both **PHGC-1** and **PHGC-1-I$_2$**, this axis is seen to lie along the long axis of the crystals (Figure S8).

- Additional data

9/ Preparation, NMR spectra and binding constant for the CB[8]•TMB-AZAP complex.

Preparation: to 0.63 mg of solid CB[8] (4.8×10^{-7} mol) were added 118 µL of a 2 mM stock solution of ***TMB-AZAP*** (2.4×10^{-7} mol) and 350 µL of D_2O for a final concentration of 1 mM of CB[8].

^{1}H NMR (300 MHz, D_2O) δ 7.58 (d, *J* = 8.7 Hz, 2H), 7.14 (d, *J* = 8.7 Hz, 2H), 6.09 (d, *J* = 8.6 Hz, 4H), 5.89 – 5.70 (m, 21H), 5.42 (s, 14H), 4.3-3.95 (m+dd, *J* = 15.2, 3.9 Hz, 18H), 3.89 (s, 3H), 3.8 (m, 2H), 3.45-3.35 (s+m, 8H), 3.28 (br m, 2H), 3.17 (br m, 6H), 2.94 (br m, 2H), 2.22 (ref acetone).

^{31}P{H} NMR (121 MHz, D_2O) δ -13.09 (s).

The exchange was slow with respect to the NMR timescale and unexpectedly, two equivalents of CB[8] were necessary to displace equilibria exclusively toward the **CB[8]•TMB-AZAP** complex leaving a fraction of insoluble CB[8].

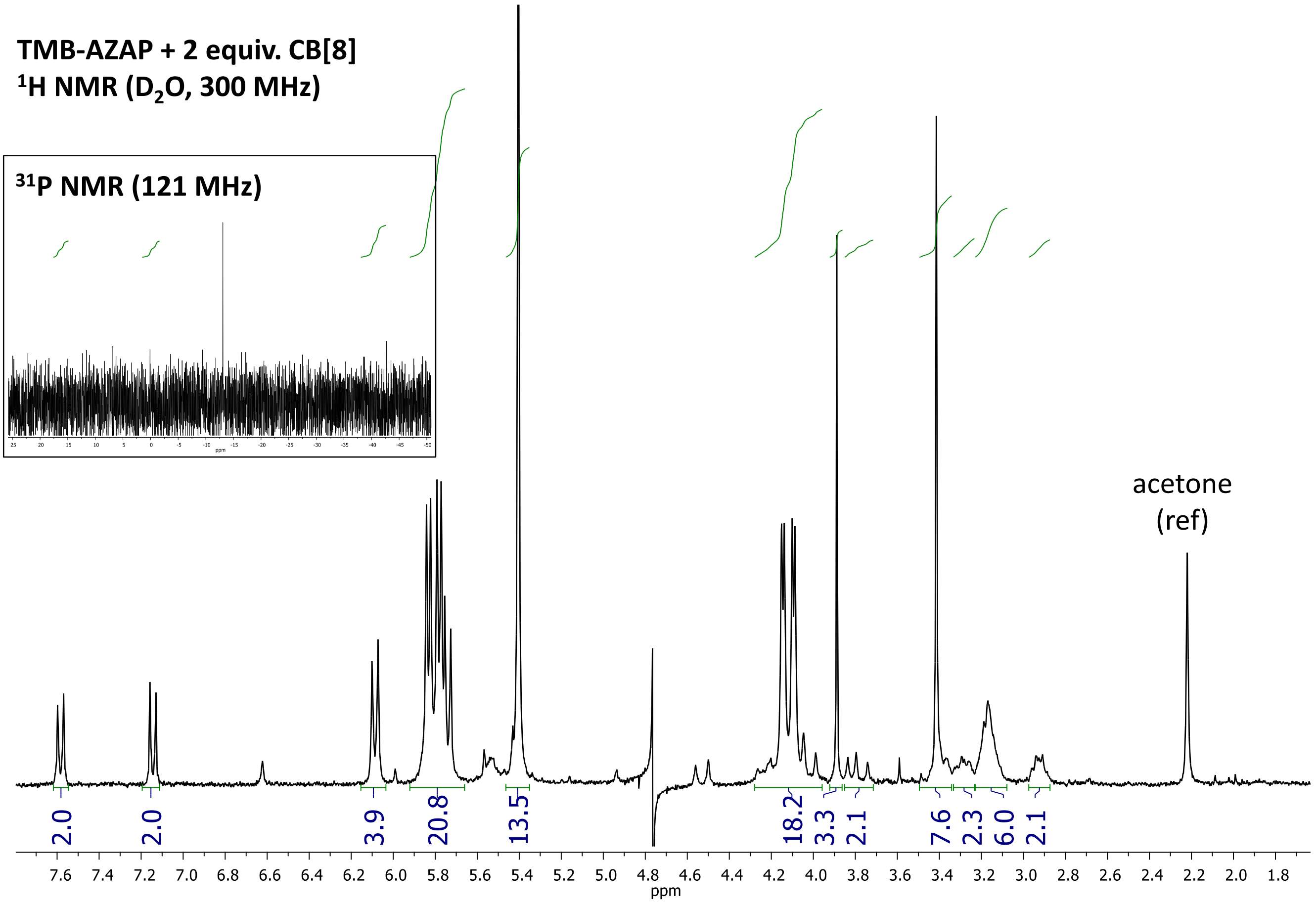

Figure S1a. ^{1}H NMR and ^{31}P{H} spectra of the CB[8]•**TMB-AZAP** complex in D_2O.

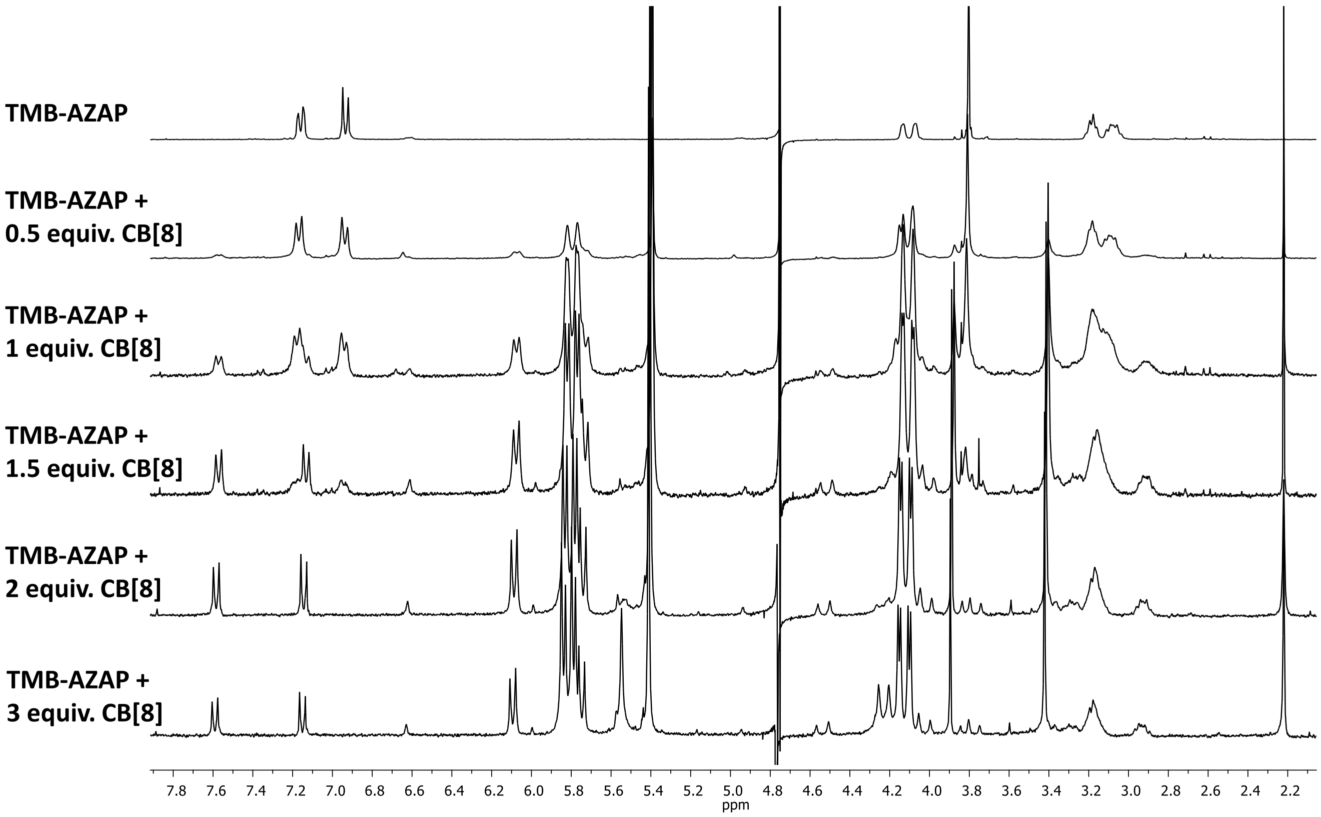

Figure S1b. ^{1}H NMR titration of **TMB-AZAP** with CB[8] in D_2O.

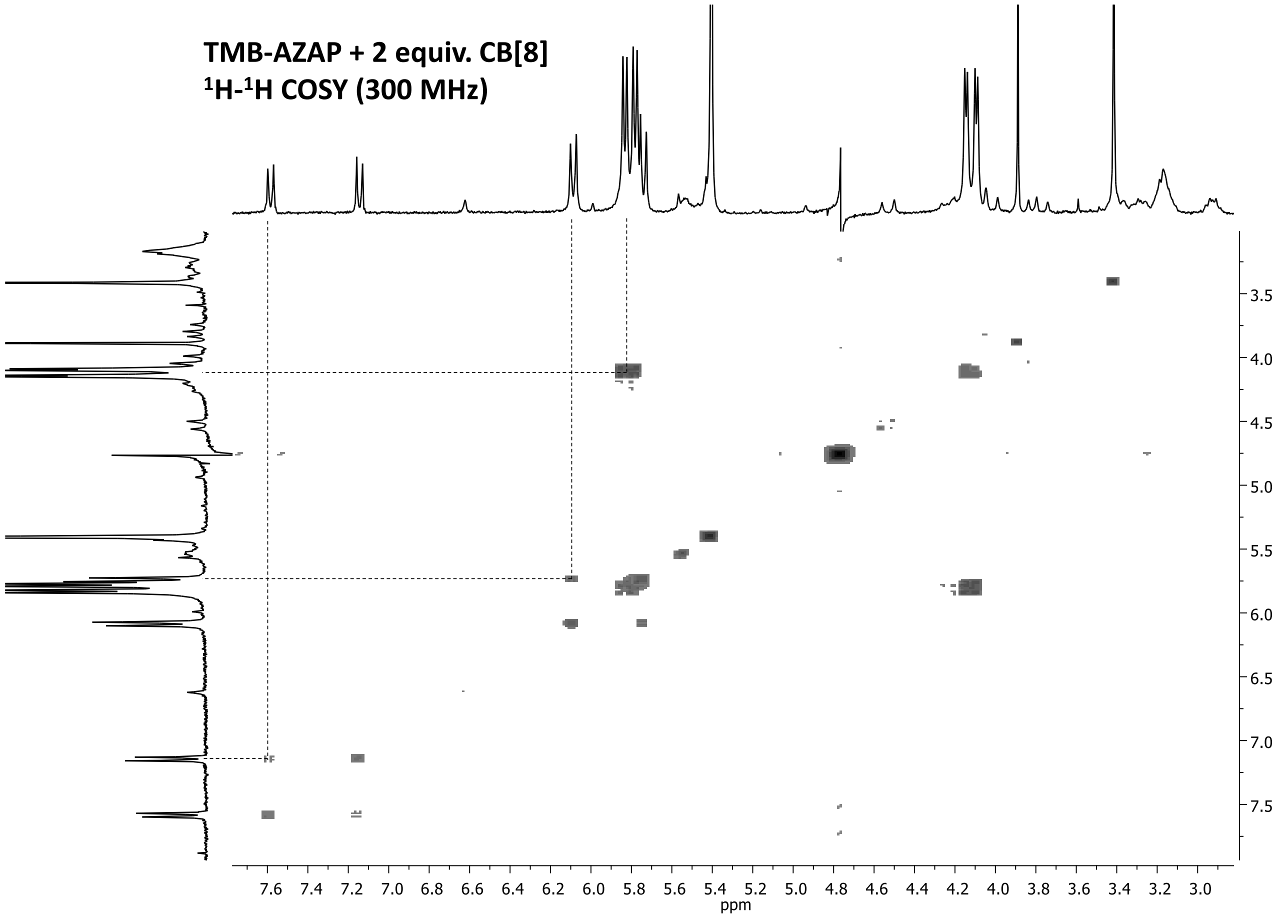

Figure S2. ^{1}H-1H COSY NMR of the CB[8]•**TMB-AZAP** complex in D_2O.

Evaluation of the binding constant for **TMB-AZAP**•CB[8] complex formation by competitive NMR.

The **TMB-AZAP** name was contracted to **AZAP** in the following for clarity. The **AZAP**•CB[8] binding constant was evaluated using a competitive ^{1}H NMR binding method, following a procedure of Macartney and co-workers.[11] NMR spectra were collected at 298 K on a Bruker AC400 (64 scans) from 1 mM solutions of **AZAP**•CB[8].
The competitor guest was dimethylviologen dichloride (**DMV**). The binding constant of **DMV**•CB[8] (1.1 × 10^5 M^{-1}) has been reported in the literature.[12]
The chemical shifts of free **DMV** and of the **DMV**•CB[8] complex were determined in D_2O from 1 mM solutions and using acetone as internal reference (2.220 ppm) (Table S1). The $\Delta\delta_{lim}$ value was determined from the subtraction of the chemical shift of free **DMV** and of **DMV**•CB[8] (to ensure the full inclusion of **DMV** in CB[8], a CB[8]:**DMV** ratio of 2:1 was applied). Since the signal of proton H_3 of **DMV** presents the highest $\Delta\delta_{lim}$, calculations of the binding constant were based using this characteristic signal. Then, ^{1}H NMR spectra of a mixture of 1 equiv of **DMV** (1 mM), 1 equiv of **AZAP** (1 mM) and 1 equiv of CB[8] (1 mM) were recorded.
According to the method of Macartney et al,[11] the binding constant of **AZAP** was calculated from the chemical shifts of proton H_3 of **DMV**•CB[8] in the competitive spectra (Figures S3) and following the equation Eq1. More precisely, we considered the following equilibria:

$$\text{AZAP} + \text{CB[8]} \leftrightarrows \text{AZAP•CB[8]} \quad K_{AZAP} = \frac{[AZAP.CB[8]]}{[AZAP]*[CB[8]]}$$

$$\text{DMV} + \text{CB[8]} \leftrightarrows \text{DMV•CB[8]} \quad K_{DMV} = \frac{[DMV.CB[8]]}{[DMV]*[CB[8]]} = 1.1 \times 10^5\ M^{-1}$$

$$\text{DMV•CB[8]} + \text{AZAP} \leftrightarrows \text{DMV} + \text{AZAP•CB[8]} \quad K = K_{AZAP}/K_{DMV} = \frac{[DMV]*[AZAP.CB[8]]}{[AZAP]*[DMV.CB[8]]}$$

$$K_{AZAP} = \frac{[DMV]*[AZAP.CB[8]]}{[AZAP]*[DMV.CB[8]]} \times K_{DMV}$$

$$[\text{AZAP•CB[8]}] + [\text{DMV•CB[8]}] = 1\ \text{mM}$$

$$[\text{DMV}] + [\text{DMV•CB[8]}] = 1\ \text{mM}$$

$$[\text{AZAP}] + [\text{AZAP•CB[8]} = 1\ \text{mM}$$

$$\Rightarrow [\text{DMV}] = [\text{AZAP•CB[8]}] \text{ and } [\text{AZAP}] = [\text{DMV•CB[8]}]$$

Table S1. Chemical shifts, % of free DMV and calculated binding constant.

solution	δ_{H3}		% of free DMV	Calculated binding constant K_{AZAP}
**DMV**	7.760 ppm	$\Delta\delta_{lim}$ = 0.746 ppm	100	-
**DMV**•CB[8]	8.506 ppm		0	-
**DMV**/CB[8]/**AZAP**	8.450 ppm[a]			1.45 × 10^7 M^{-1}

[a] average of 3 experiments

% of free **DMV** in competitive solution = 100 × (8.430-7.760)/$\Delta\delta_{lim}$ = 92%
% of **DMV**•CB[8] in competitive solution = 100 × (8.506-8.410)/$\Delta\delta_{lim}$ = 8%

Eq1 $K_{AZAP} = K_{DMV} \times (92)^2 /(8)^2 = 145 \times 10^5\ M^{-1}$

This method permitted to estimate a **AZAP**•CB[8] binding constant of 1.45 × 10^7 M^{-1}.

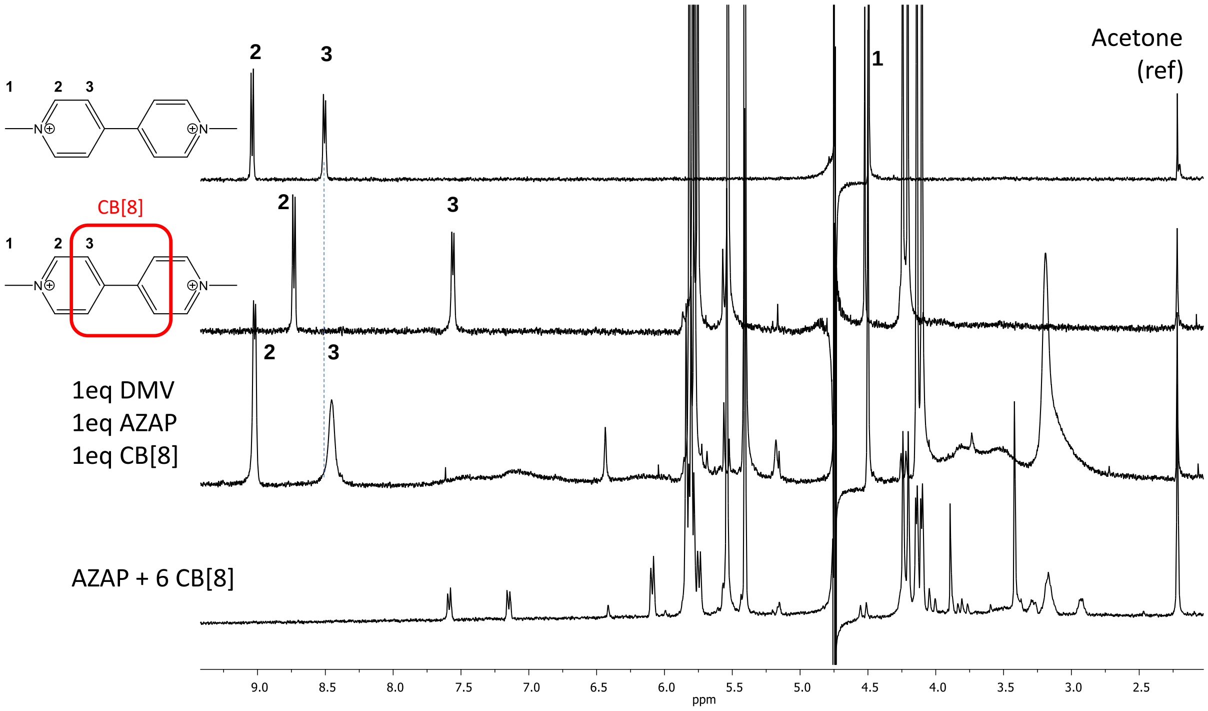

Figure S3. ^{1}H NMR spectra (from top to bottom) of free **DMV**, **DMV**•CB[8], of competitive solution of **DMV**/**AZAP**/CB[8] (ratio 1/1/1, 1 mM) and of **AZAP** in the presence of 6 equiv of CB[8] (D_2O, 400 MHz, NS = 64, 300 K, acetone as internal reference δ = 2.220 ppm).

Preparation of competitive solution: 0.75 mg of solid CB[8] (5.6 10^{-7} mol), 56 µL of a 10 mM solution of **DMV** (5.6 10^{-7} mol), 282 µL of 2 mM solution of **AZAP** (5.6 10^{-7} mol) and 230 µL of D_2O were mixed and the solution was analyzed by ^{1}H NMR (400 MHz, 300 K, NS = 64).

10/ Figures and table from the CB[8]•TMB-AZAP (PHGC-1) crystal structure

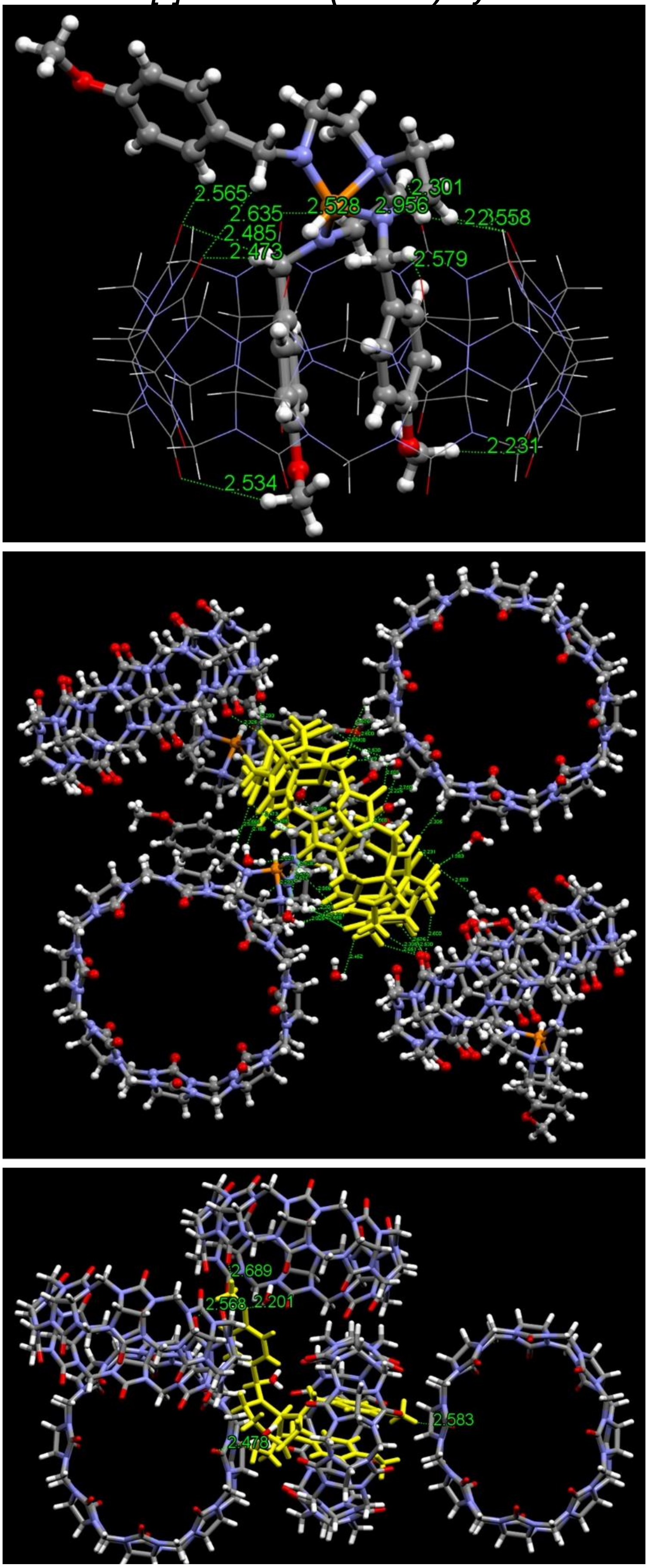

Figure S4. Host:guest interactions (top), and interactions of CB[8] toward nearest neighbours (middle) and interactions of **TMB-AZAP** toward nearest neighbours (bottom).

CB[8] interacts with 4 other neighboring macrocycles, 1 guest inside its cavity and 2 guests outside and 6 water molecules (2 outside, and two with each CB[8] carbonyl rim). **TMP-AZAP** interacts with 1 CB[8] strongly (host:guest complex) and 4 other CB[8] by CH•••O hydrogen bonds, and 1 water molecule.

Table S2. Selected parameters from the crystal structure of CB[8]•**TMB-AZAP**.

**Compound**	CB[8]•**TMB-AZAP**
CCDC number	2143333
Formula	$C_{30}H_{40}N_4O_3P$, $C_{48}H_{48}N_{32}O_{16}$, 6.5(H_2O)
M_w	1981.91
Crystal system	trigonal
Temp./ K	295
Space group	*R*3
a/ Å	39.8566(2)
c/ Å	17.01570(10)
V/ Å^3	23408.9 (3)
Z	9
Dc/g.cm^{-3}	1.265
Crystal colour	colourless
Crystal size/mm^3	0.38*0.28*0.22
μ(Cu-Kα)/mm^{-1}	1.54184
N° of refl. measured	36124
N° of obs. refl.[$F^2 > 4\sigma F^2$]	15598
N° parameters refined	1298
N° restraints	50
R_1 [F^2>4σF^2]	0.0726
wR_1 [F^2>4σF^2]	0.2012
R_2 [all refl.]	0.0764
wR_2 [all refl.]	0.2127
Goodness of fit [all refl.]	1.027
Residual Fourier/e. Å^{-3}	-0.39; 0.48
Flack	0.08(3)

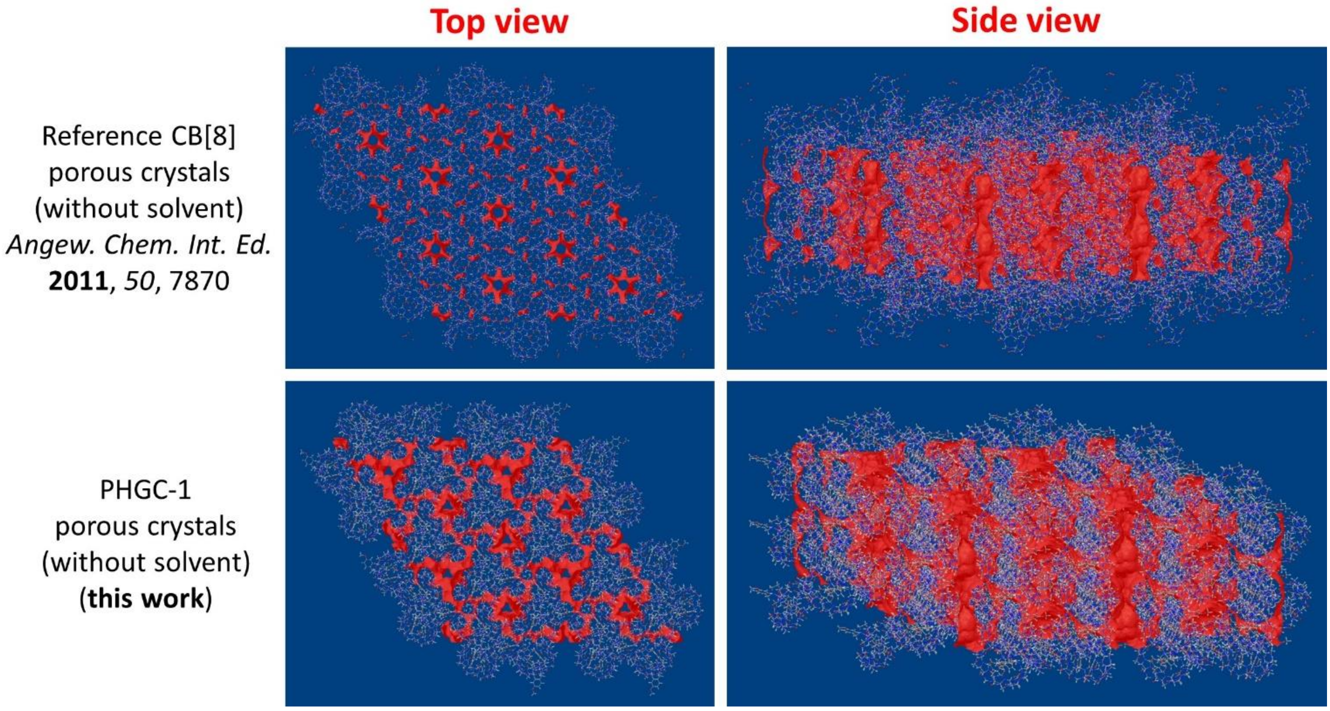

Figure S5. Comparison between the shape of channels[7] of the previously reported CB[8] hydrate structure (top line) and of the shape of channels of PHGC-1 (bottom line).

11/ TGA analysis of PHGC-1

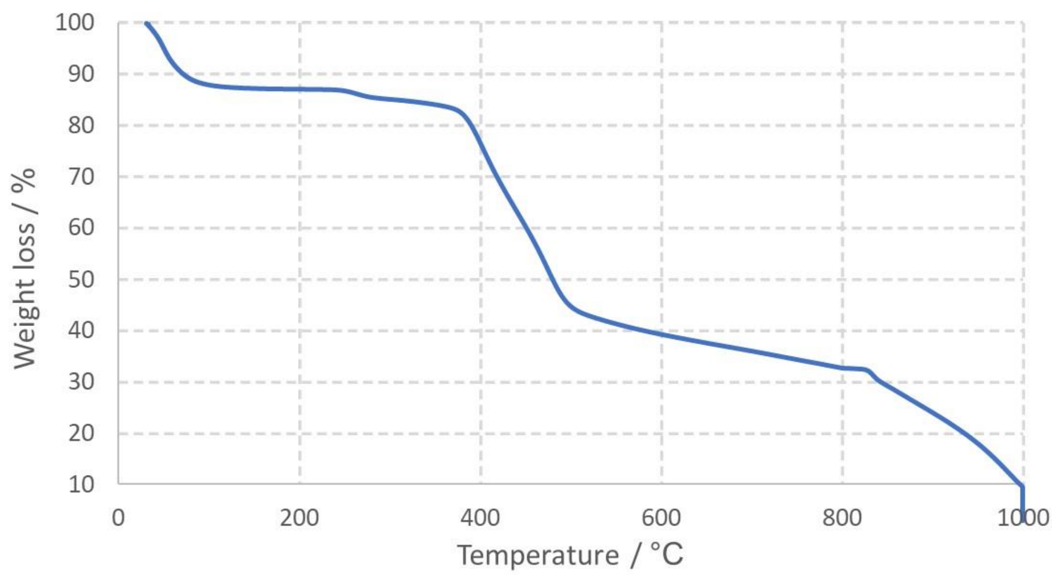

Figure S6. TGA analysis of (hydrated or as-prepared) CB[8]•**TMB-AZAP** (**PHGC-1**) single crystals.

12/ Elemental analysis of PHGC-1 and PHGC-1•I_2.

1CB[8], 1**TMB-AZAP**, 22 H_2O: Calc. % (Found %): C 40.79 (40.17), H 5.79 (5.37), N 21,96 (21.78).
1CB[8], 1**TMB-AZAP**, 1H_2O, 4.3 I_2: Calc. % (Found %): C 31.13 (30.85), H 3.01 (3.25), N 16.75 (16.69).

13/ Figures and table from the CB[8]•TMB-AZAP•I_2 (PHGC-1)•I_2 crystal structure

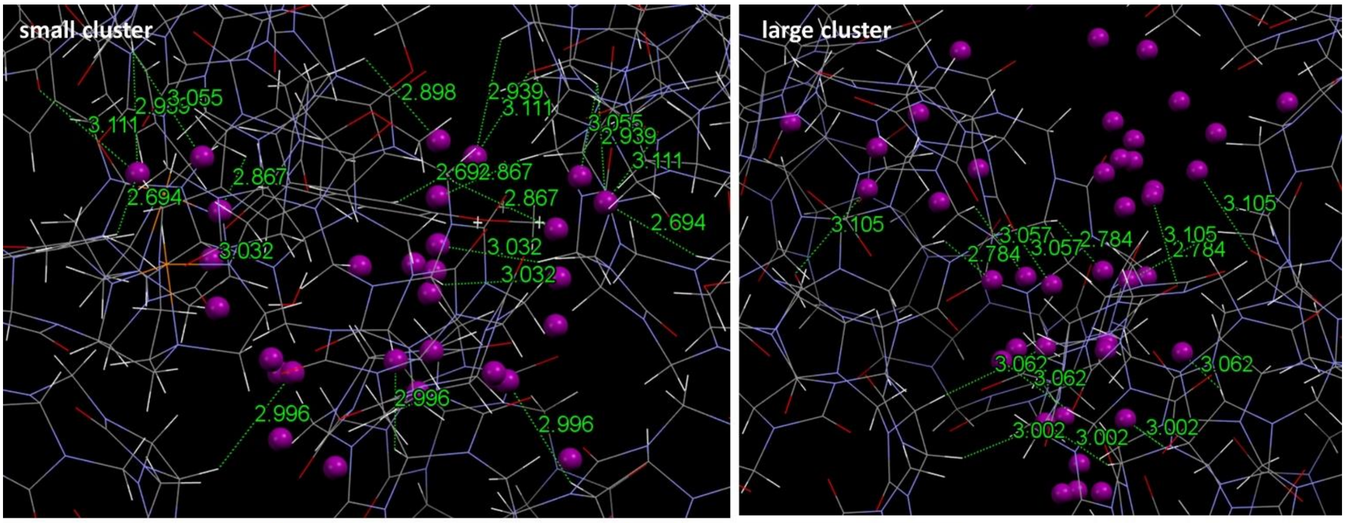

I2		small cluster		large cluster	
d (CH•••I)/Å	d (CO•••I)/Å	d (CH•••I)/Å	d (CO•••I)/Å	d (CH•••I)/Å	d (CO•••I)/Å
2.62	2.89	3.00	3.11	3.00	3.11
2.80	2.92	3.00	3.11	3.00	3.11
	2.69	3.00	3.11	3.00	3.11
		2.69		3.06	
		2.69		3.06	
		2.69		3.06	
cut-off	cut-off	3.03		2.78	
d (H•••I)/Å	d (O•••I)/Å	3.03		2.78	
3.2 Å	3.5 Å	3.03		2.78	
		2.87		3.06	
		2.87		3.06	
		2.87		3.06	
		2.94			
		2.94			
		2.94			
		3.06			
		3.06			
		2.90			

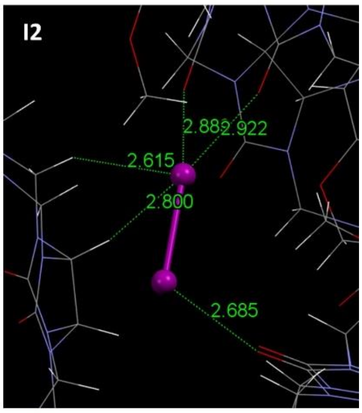

Figure S7. Hydrogen and halogen bonds in the **PHGC-1•I_2** single crystals.

Table S3. Selected parameters from the crystal structure of CB[8]•**TMB-AZAP**•I_2.

**Compound**	CB[8]•**TMB-AZAP•I₂**
CCDC number	2143334
Formula	$C_{30}H_{40}N_4O_3P$, $C_{48}H_{48}N_{32}O_{16}$,H_2O, 4.783I
M_w	2515.21
Crystal system	trigonal
Temp./ K	295
Space group	*R*3
a/ Å	39.6704(5)
c/ Å	16.8020(2)
V/ $Å^3$	22899.4 (6)
Z	9
Dc/g.cm^{-3}	1.642
Crystal colour	Red
Crystal size/mm^3	0.22*0.18*0.08
μ(Cu-Kα)/mm^{-1}	1.54184
N° of refl. measured	140970
N° of obs. refl.[$F^2 > 4\sigma F^2$]	14709
N° parameters refined	1436
N° restraints	266
R_1 [$F^2>4\sigma F^2$]	0.1033
wR_1 [$F^2>4\sigma F^2$]	0.2777
R_2 [all refl.]	0.1131
wR_2 [all refl.]	0.2950
Goodness of fit [all refl.]	1.219
Residual Fourier/e. Å^{-3}	-0.567; 1.368
Flack	0.039(11)

14/ Variation of the diffusion coefficient of water as a function of I_2 diffusion

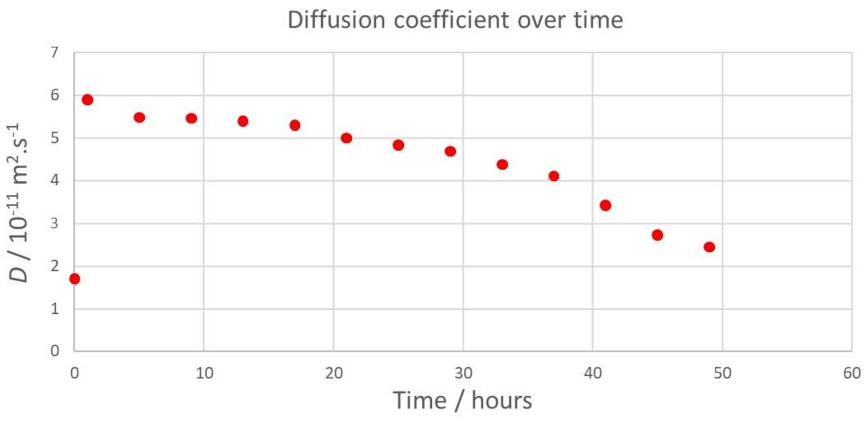

Figure S8. Evolution of the diffusion coefficient of water as a function of time.

15/ *Results of Second Harmonic Generation.*

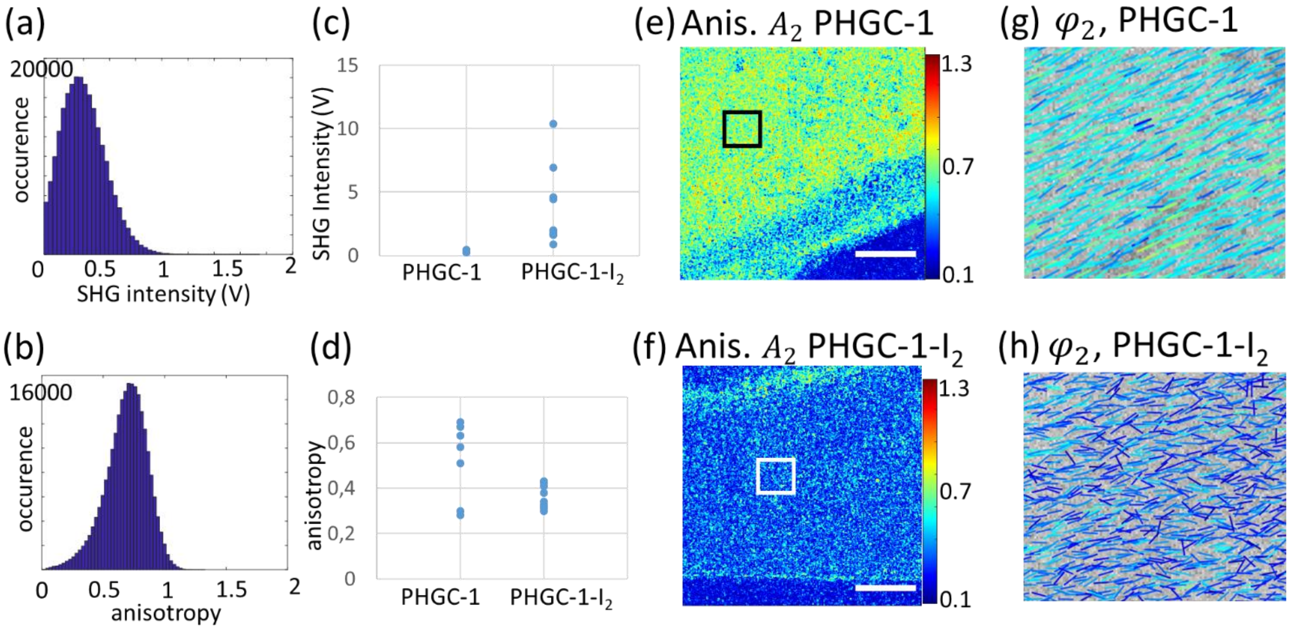

Figure S9. (a,b) Typical SHG intensity (V) and anisotropy A_2 measured in a **PHGC-1** crystal. (c,d) Values reported over 6 regions (4 different crystals) in both **PHGC-1** and **PHGC-1-I$_2$** crystals. (e) Anisotropy A_2 map measured in a **PHGC-1** crystal. (f) Anisotropy A_2 map measured in a **PHGC-1-I$_2$** crystal. Scale bars: 20 μm. (g,h) Map of the orientation angle φ_2 measured in both crystal types (zoom on the square shown in (e,f)). The sticks orientation relative to the horizontal direction correspond to the retrieved φ_2 angle, while the color of the sticks corresponds to the measured A_2 at the pixel location.

16/ *UV-vis spectrum of PHGC-1-I$_2$ crystals.*

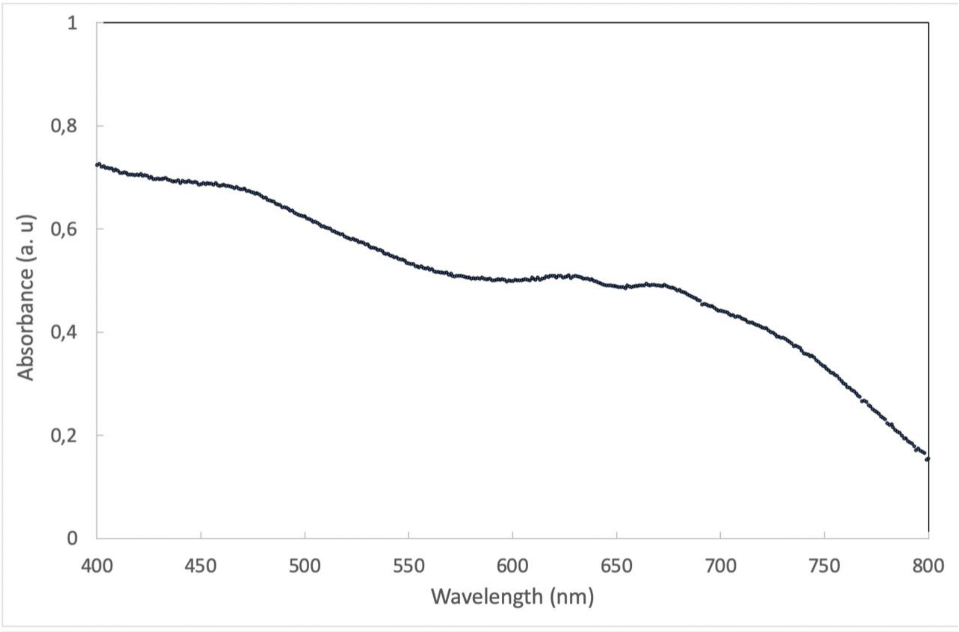

Figure S10. Solid-state UV-vis spectrum of **PHGC-1-I$_2$** crystals.

17/ Iodine uptake from solution by PHGC-1 crystals.

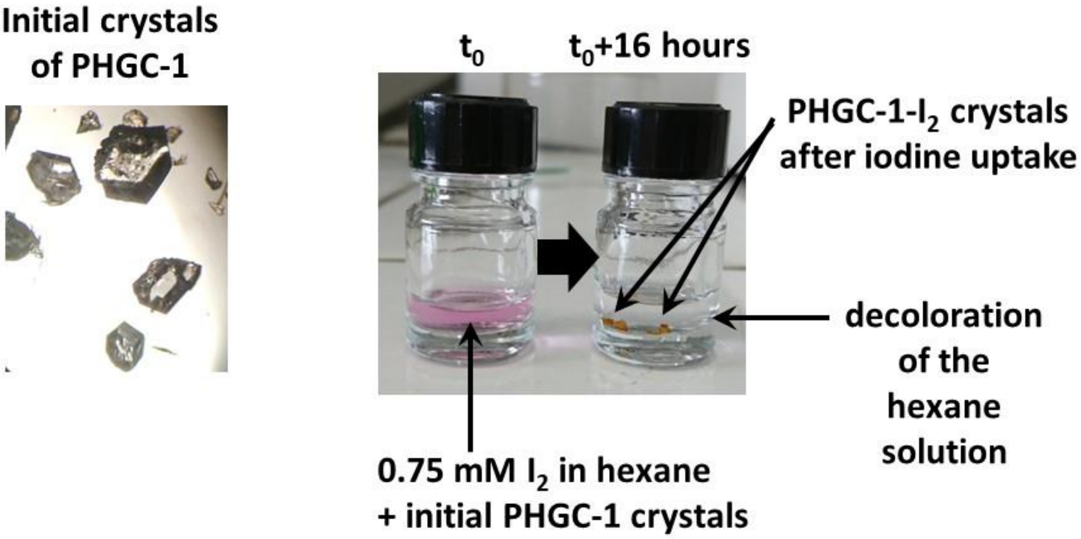

Figure S11. Iodine uptake from a hexane solution by **PHGC-1** crystals.

18/ References.

[1] D. Bardelang, K. A. Udachin, D. M. Leek, J. C. Margeson, G. Chan, C. I. Ratcliffe, J. A. Ripmeester, *Cryst. Growth Des.* **2011**, *11*, 5598–5614.
[2] P. D. Raytchev, A. Martinez, H. Gornitzka, J.-P. Dutasta, *J. Am. Chem. Soc.* **2011**, *133*, 2157–2159.
[3] D. H. Wu, A. D. Chen, C. S. Johnson, *J. Magn. Reason. Ser. A.* **1995**, *115*, 260–264.
[4] Jmol: an open-source Java viewer for chemical structures in 3D. http://www.jmol.org/.
[5] AMPAC 11, 1992-2017 Semichem, Inc. 12456 W 62nd Terrace - Suite D, Shawnee, KS 66216.
[6] J. J. P. Stewart. *J. Mol. Model.* **2007**, *13*, 1173–1213.
[7] O. V. Dolomanov, L. J. Bourhis, R. J. Gildea, J. A. K. Howard, H. Puschmann, *J. Appl. Cryst.* **2009**, *42*, 339–341.
[8] G. M. Sheldrick, *Acta Cryst.* **2015**, *A71*, 3–8.
[9] G. M. Sheldrick, *Acta Cryst.* **2015**, *C71*, 3–8.
[10] C. Rendon-Barraza, F. Timpu, R. Grange, S. Brasselet. *Sc. Reports* **2019**, *9*, 1670.
[11] M. A. Gamal-Eldin, D. M. Macartney, *Org. Biomol. Chem.* **2013**, *11*, 1234–1241.
[12] W. S. Jeon, H.-J. Kim, C. Lee, K. Kim, *Chem. Commun.* **2002**, 1828–1829.
